# Catastrophic consequences of prolonged immobilization after intracerebral hemorrhage: a case report of crush syndrome combined with capillary leak syndrome

**DOI:** 10.3389/fnins.2026.1889845

**Published:** 2026-07-23

**Authors:** Pengpeng Li, Yangyang Gao, Junfeng Li, Shaohua Lin, Zehong Zhang, Lei Luo, Wei Liu

**Affiliations:** Department of Neurosugery Xi'an Aerospace Hospital of Northwest University, Xi'an, Shanxi, China

**Keywords:** acute kidney injury, capillary leak syndrome, case report, central venous pressure, crush syndrome, intracerebral hemorrhage, rhabdomyolysis

## Abstract

**Background:**

Spontaneous intracerebral hemorrhage is a neurological emergency with high mortality and disability rates. Prolonged immobilization after stroke can trigger severe systemic complications. The simultaneous occurrence of crush syndrome and capillary leak syndrome in the setting of acute intracerebral hemorrhage is extremely rare and life-threatening, and no established treatment protocol exists.

**Case summary:**

A 64-year-old man with hypertension was found unconscious on his home floor. The estimated time from onset to discovery exceeded 24 h. He was diagnosed with left basal ganglia intracerebral hemorrhage of approximately 5.6 mL with intraventricular extension. On admission, he was comatose with a Glasgow Coma Scale score of 9. Systemic involvement was extensive: severe bilateral leg swelling with multiple tension bullae, acute kidney injury requiring continuous renal replacement therapy, severe rhabdomyolysis with a peak creatine kinase exceeding 50,000 U/L, hyperkalemia, with an initial arterial blood gas in the emergency department showing 6.9 mEq/L, elevated liver enzymes, and distributive shock. Admission central venous pressure was negative, strongly indicating severe hypovolemia. Chest CT already showed hypostatic pneumonia. The clinical presentation was consistent with crush syndrome from prolonged compression, which then led to secondary capillary leak syndrome. The patient underwent emergency neuroendoscopic hematoma evacuation and external ventricular drainage. Postoperatively, he developed atrial fibrillation and deep vein thrombosis. After 3 weeks of multidisciplinary intensive care, his liver and kidney function gradually improved, and the hypostatic pneumonia was controlled. He survived to discharge with a Glasgow Coma Scale score of 11, left limb muscle strength grade 3/5, and right limb muscle strength grade 0/5, and was transferred to a local hospital for continued rehabilitation. At 3-month follow-up, he was walking independently (mRS 2), with significant improvements in neurological function and quality of life.

**Conclusion:**

This case reveals a frequently overlooked but deadly clinical pathway: prolonged immobilization after intracerebral hemorrhage can lead to traumatic rhabdomyolysis, crush syndrome, and secondary capillary leak syndrome. A negative central venous pressure, a rare finding, provided supportive evidence of severe hypovolemia due to capillary leak syndrome. This clinical triad represents a unique high-risk condition that demands a shift in treatment philosophy from neurocentric care to systemic resuscitation.

## Introduction

Intracerebral hemorrhage accounts for 10%–15% of strokes in high-income countries and 25%–55% in low- and middle-income countries (Li et al., 2024). It carries high rates of mortality and disability (Li et al., 2025). Early treatment focuses on hematoma evacuation, intracranial pressure control, and prevention of secondary neurological injury (Puy et al., 2025). However, an important contributor to poor outcomes after intracerebral hemorrhage comes from non-neurological systemic complications.

Prolonged immobilization, especially when a fall is unwitnessed, can trigger a series of systemic problems. The most serious of these is crush syndrome, where traumatic rhabdomyolysis releases myoglobin, potassium, phosphorus, and other substances into the bloodstream, causing acute kidney injury, arrhythmias, and metabolic acidosis (Usuda et al., 2023). Local signs of crush syndrome include swelling, tension bullae, and skin bruising at the compression sites, which reflect the severity of muscle necrosis and interstitial fluid accumulation (Akrivos et al., 2025). Another less recognized but equally dangerous consequence is capillary leak syndrome, characterized by increased capillary permeability. Plasma leaks from the intravascular space into the tissue spaces, leading to severe hypotension, hemoconcentration, and generalized edema (Druey et al., 2024). A negative central venous pressure is a rare but clinically significant finding. When interpreted in the appropriate context, it serves as an objective indicator of severe intravascular volume depletion, providing a valuable basis for clinical diagnosis. In addition, hypostatic pneumonia from prolonged bed rest is a common early complication after stroke and can itself trigger or worsen systemic inflammation (de Jonge et al., 2022). The simultaneous presentation of acute intracerebral hemorrhage, crush syndrome, secondary capillary leak syndrome, and hypostatic pneumonia is extremely rare. The overlapping clinical features, including hypotension, edema, and acute kidney injury, pose major challenges for diagnosis and treatment. This report details the successful management of this deadly triad and describes the patient’s favorable recovery after discharge, offering important clinical lessons.

## Case presentation

A 64-year-old right-handed man with a 2-year history of hypertension (medications unknown) was brought to the emergency department after being found lying on his home floor. The time from when he was last seen normal was estimated to be over 24 h. He had no history of diabetes, coronary artery disease, smoking, or alcohol use. Physical examination revealed severe swelling of both legs with multiple tension bullae. A few bullae were intact, but most had ruptured, exposing pink bases with minimal serous drainage. Extensive bruising and edema were also present in both legs (Figure 1A).

**Figure 1:**
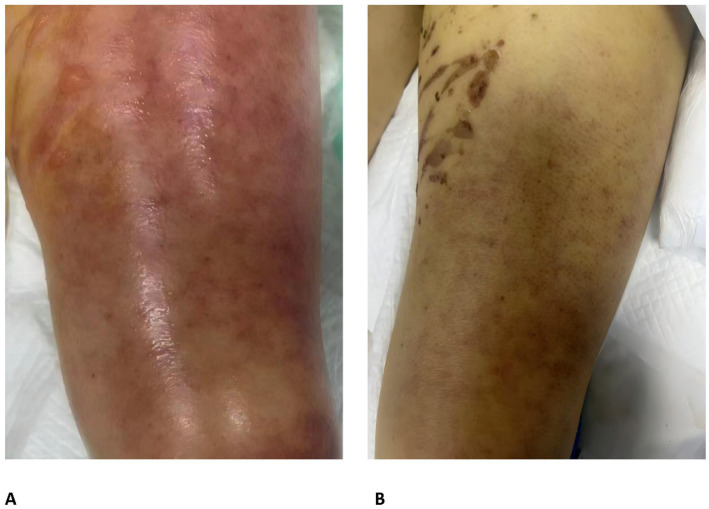
**(A)** On admission, showing severe bilateral leg swelling, extensive ecchymosis, and multiple tension bullae, indicating severe soft tissue compression and muscle necrosis. **(B)** After 3 weeks of treatment including continuous renal replacement therapy and supportive wound care, the lower extremity edema had significantly subsided and the bullae healed with re-epithelialization, without signs of secondary infection.

On day 0 at 01:45 a.m., he was comatose with a Glasgow Coma Scale score of 9. Vital signs: blood pressure 91/70 mmHg, heart rate 133 beats per minute, respiratory rate 22 breaths per minute, temperature 36.4 °C. Central venous pressure monitoring showed a negative value, an extremely rare clinical finding that strongly suggested severe intravascular volume depletion, consistent with the pathophysiology of massive fluid extravasation from capillary leak syndrome into the tissue spaces. Laboratory tests: serum potassium 6.06 mEq/L, serum creatinine 284 μmol/L, creatine kinase 45,810 U/L, lactate dehydrogenase 1,696 U/L, and procalcitonin (PCT) 1.63 ng/mL. Imaging studies: head CT showed a 5.6 mL left basal ganglia intracerebral hemorrhage with intraventricular extension and obstructive hydrocephalus; head CTA showed intracranial atherosclerosis; chest CT showed bilateral hypostatic pneumonia, interstitial changes, and possible endobronchial mucus plugs; abdominal CT showed gallstones and bilateral adrenal thickening.

Initial diagnoses included: left basal ganglia intracerebral hemorrhage with intraventricular extension, secondary intraventricular hemorrhage, obstructive hydrocephalus, grade 3 hypertension at very high risk; hypostatic pneumonia; crush syndrome diagnosed based on creatine kinase greater than 50,000 U/L with acute kidney injury and bilateral lower extremity tension bullae; capillary leak syndrome diagnosed based on distributive shock, generalized edema, hemoconcentration, hypoalbuminemia, and negative central venous pressure; along with hyperkalemia, acute kidney injury, and acute liver injury. At 06:00 a.m. on day 0, the patient underwent emergency neuroendoscopic intracerebral hematoma evacuation, right external ventricular drainage, and intracranial pressure monitor placement. The surgery went smoothly with 200 mL of blood loss. Preoperative head CT showed a left basal ganglia hematoma of approximately 5.6 mL with intraventricular extension as shown in Figure 2A; postoperative day 1 head CT showed near complete hematoma evacuation, reduced intraventricular blood, and a well positioned external ventricular drain as shown in Figure 2B.

**Figure 2:**
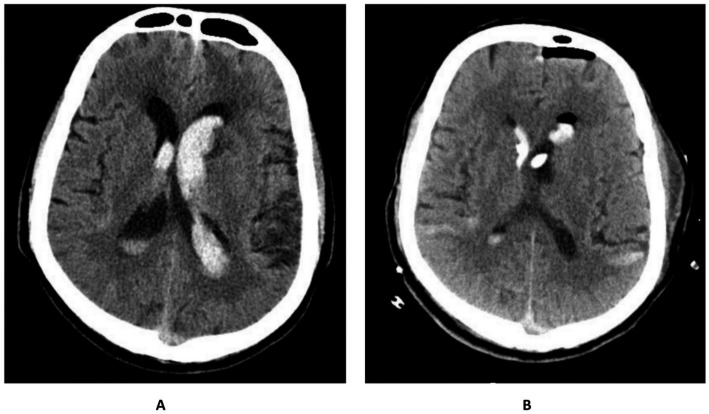
**(A)** Preoperative non-contrast head CT showing a left basal ganglia hematoma of approximately 5.6 mL with intraventricular extension. **(B)** Postoperative day 1 non-contrast head CT showing near-complete evacuation of the hematoma, reduced intraventricular blood, and a well-positioned external ventricular drain.

Postoperatively, the patient remained sedated for intracranial pressure control and neuroprotection, during which the GCS score decreased to a nadir of 6. As sedation was gradually tapered from Day 4 onward, his level of consciousness steadily improved, reaching a GCS of 11 at discharge.

After surgery, his condition worsened. He became anuric. The peak creatine kinase level exceeded 50,404 U/L. Daily continuous renal replacement therapy was started on day 1 and continued for 14 days to manage severe acute kidney injury and hyperkalemia, with a maximum serum potassium of 6.24 mEq/L. The initiation, duration, and dosage of CRRT were adjusted individually based on the patient’s renal function recovery, hemodynamic stability, and fluid balance. Fluid management was guided by trends in central venous pressure and hourly urine output, combined with other hemodynamic parameters, rather than a fixed preset protocol. He developed distributive shock requiring vasopressors. On day 3, new onset rapid atrial fibrillation developed with a heart rate of 160 beats per minute, which was managed with esmolol. The hypostatic pneumonia present on admission was confirmed by sputum culture as *Staphylococcus aureus* and *Corynebacterium striatum* infection. He was treated with piperacillin tazobactam, along with enhanced airway care, regular suctioning, and positioning to promote secretion drainage.

Management of the lower extremity tension bullae focused on protecting intact bullae from rupture, debriding and covering ruptured bullae with sterile dressings, elevating the limbs to promote venous return, and monitoring for signs of infection. After 3 weeks of treatment, the lower extremity edema had significantly subsided and the bullae healed with re-epithelialization as shown in Figure 1B. On day 12, vascular ultrasound showed a left calf muscle vein thrombosis, and rivaroxaban was started. Lower extremity and scrotal edema gradually improved, and compartment syndrome was repeatedly ruled out by physical examination. Sedation was maintained for 10 days after surgery. Intracranial pressure remained normal. The Glasgow Coma Scale score gradually improved from 6 to 11. At discharge, his Glasgow Coma Scale score was 11. He could open his eyes spontaneously and follow simple commands. He had significant neurological deficits: left limb muscle strength grade 3/5 and right limb muscle strength grade 0/5, with a modified Rankin Scale (mRS) score of 5.

On day 24, his vital signs were stable, and liver and kidney function had improved significantly. Serum creatinine fell from a peak of 478 to 242 μmol/L, and alanine aminotransferase fell from 263 to 54 U/L. He was transferred to a local hospital for continued rehabilitation. A telephone follow up 3 months after discharge revealed that he was walking independently, with significant recovery of daily living activities and no further seizures or cerebrovascular events. His mRS score improved to 2 at 3-month follow-up (Table 1).

**Table 1:** Dynamic changes in key laboratory parameters.

Parameter	Day 0	Day 1	Day 3	Day 12	Day 21	Reference range
Creatine kinase U/L	45,810	50,404	34,169	1,404	191	24–195
Serum creatinine μmol/L	284	462	478	423	242	53–115
Serum potassium mEq/L	6.06	6.24	5.9	4.1	3.8	3.5–5.1
ALT U/L	268	260	263	92	54	<50
AST U/L	1,135	1,128	947	466	48	<50
D-dimer μg/mL	21.9	4.7	7.5	14.9	10.0	<0.5
24 h urine output mL	53	136	150	1,150	2,200	>800

## Discussion

This case demonstrates the devastating systemic consequences of prolonged immobilization after an acute neurological event, as well as the good outcomes that can be achieved with aggressive multidisciplinary treatment and subsequent rehabilitation. This patient’s presentation was not simply intracerebral hemorrhage with pneumonia. It was a unique clinical entity: left basal ganglia hemorrhage with intraventricular extension leading to crush syndrome complicated by secondary capillary leak syndrome, with hypostatic pneumonia present on admission as an additional inflammatory insult. The finding of a negative central venous pressure on admission provided supportive evidence of severe hypovolemia due to capillary leak syndrome.

The initial event was loss of consciousness from the left basal ganglia hemorrhage with intraventricular extension, which caused the patient to lie on a hard floor for a prolonged period. The intraventricular blood worsened the elevated intracranial pressure and the degree of consciousness impairment, preventing him from changing position. Prolonged compression of the back, buttocks, and lower limb muscle groups led to traumatic rhabdomyolysis, releasing myoglobin, potassium, phosphorus, and creatine kinase into the bloodstream. The acute kidney injury in this case fits the classic mechanism of crush syndrome, driven by myoglobin cast nephropathy, tubular obstruction, oxidative stress injury, and reduced kidney perfusion (Miyauchi et al., 2024). The tension bullae on the lower limbs are a characteristic local sign of crush syndrome. They form when muscle necrosis and capillary damage cause massive fluid accumulation in the tissues, and the increased osmotic pressure draws fluid into the subepidermal layer. The presence of bullae indicates severe compression and widespread muscle necrosis (Grivei et al., 2020).

Regarding capillary leak syndrome, the systemic inflammatory response triggered by severe trauma including rhabdomyolysis and intracerebral hemorrhage releases large amounts of cytokines, including interleukin 1, interleukin 6, and tumor necrosis factor alpha (Siddall et al., 2017). These cytokines increase capillary permeability by directly damaging vascular endothelial cell junctions and inducing mediators such as vascular endothelial growth factor. This leads to the classic triad of capillary leak syndrome: hypotension, hemoconcentration, and generalized edema (Basile, 2025). A negative central venous pressure is a rare objective hemodynamic finding in capillary leak syndrome. Normally, central venous pressure ranges from 0 to 8 mmHg. A negative value is extremely rare and indicates severely reduced intravascular effective volume. In capillary leak syndrome, because large amounts of plasma leak from the intravascular space into the tissue spaces, the effective circulating volume decreases dramatically, causing the central venous pressure to drop significantly or even become negative. This finding provides an important basis for differential diagnosis: in a patient with distributive shock and generalized edema, a negative central venous pressure points clearly to capillary leak syndrome rather than cardiogenic or obstructive shock.

Hypostatic pneumonia played a significant role in this case. The patient’s impaired consciousness from the intracerebral hemorrhage and prolonged bed rest led to aspiration and secretion accumulation, resulting in hypostatic pneumonia that was already present on admission. The lung infection itself triggered systemic inflammatory response syndrome, releasing large amounts of inflammatory mediators that added to the cytokine storm from crush syndrome, further worsening capillary leak and systemic inflammation. Thus, this case actually involved a triple hit: the initial intracerebral hemorrhage, crush syndrome, and hypostatic pneumonia.

It is worth noting that capillary leak syndrome and crush syndrome worsened each other in this case. The large amount of inflammatory mediators released from crush syndrome directly triggered capillary leak. In turn, the hypovolemia and poor tissue perfusion caused by capillary leak syndrome worsened the existing kidney injury and made it more difficult to clear myoglobin from the rhabdomyolysis. The severe local tissue damage reflected by the tension bullae meant that even larger amounts of inflammatory mediators were released into the bloodstream, and the hypostatic pneumonia acted as a persistent infection source, constantly releasing inflammatory mediators that made the capillary leak difficult to reverse. This multi-loop positive feedback explains why the patient’s condition rapidly deteriorated into multi-organ failure in a short period of time.

Central venous pressure is an important hemodynamic parameter for assessing a patient’s volume status and right heart function. The normal range is typically 0 to 8 mmHg (Lloyd-Donald et al., 2025). The negative central venous pressure in this case had several important clinical implications. First, it provided supportive evidence of severe hypovolemia due to capillary leak syndrome, going beyond clinical judgment alone. Second, it helped with differential diagnosis: in a patient with both hypotension and significant generalized edema, a negative central venous pressure ruled out cardiogenic or obstructive shock and clarified that hypovolemia was the dominant feature of the distributive shock. Third, it guided treatment decisions: a negative central venous pressure strongly suggests that large volume crystalloid infusion should be avoided, because fluid will rapidly leak into the tissue spaces rather than effectively increasing circulating volume. Instead, vasopressors should be used to maintain perfusion pressure, and continuous renal replacement therapy should be started as early as possible for precise volume management. Fourth, it can serve as a monitor of treatment response: as capillary leak is controlled and intravascular volume recovers, the central venous pressure should gradually return to the normal range.

Although hypostatic pneumonia was present on admission, several factors argue against septic shock as the primary driver of hypotension. The patient had no fever, PCT was only 1.63 ng/mL, and shock preceded microbiological confirmation of infection. The pneumonia was more likely due to aspiration of vomitus. These findings, together with negative central venous pressure and generalized edema, support capillary leak syndrome as the primary cause of distributive shock, with pneumonia serving as an inflammatory amplifier.

The patient’s neurological deficits at discharge, left limb strength grade 3/5 and right limb strength grade 0/5, were consistent with the imaging findings of left basal ganglia hemorrhage with intraventricular extension. Left basal ganglia hemorrhage primarily affects right sided motor function, so the right limb strength of 0/5, complete hemiplegia, was expected. The milder impairment on the left side, grade 3/5, may have resulted from mass effect on surrounding tissue or bilateral effects of elevated intracranial pressure. The fact that he was walking independently at 3 month follow up suggests that neuroplasticity and rehabilitation played important roles. Despite experiencing multiple systemic insults including crush syndrome, capillary leak syndrome, and acute kidney injury, his neurological function still recovered remarkably, indicating that timely control of systemic complications is equally important for neurological outcomes.

A search of the PubMed database from 2000 to 2024 found no case reports of crush syndrome after stroke. In the existing literature, crush syndrome is mainly reported after traumatic events such as earthquakes, building collapses, and traffic accidents. This appears to be the first case report of acute intracerebral hemorrhage complicated by both crush syndrome and secondary capillary leak syndrome. It is also the first to systematically describe the complete clinical features and pathophysiology of secondary capillary leak syndrome in the setting of post-hemorrhage crush syndrome, and the first to detail the clinical significance of a negative central venous pressure in capillary leak syndrome and how it guides treatment decisions.

Several key clinical lessons can be drawn from this case. First, for any patient found unconscious after a fall, especially when the estimated down time exceeds 6 h, a full systemic evaluation is essential, not just a neurological one. This evaluation should include checking for muscle tenderness and swelling to assess rhabdomyolysis risk, examining skin integrity for tension bullae or pressure sores, measuring kidney function and electrolytes, checking creatine kinase levels, monitoring central venous pressure, and obtaining chest imaging to evaluate for hypostatic pneumonia. The presence of tension bullae suggests severe compression and a high risk of crush syndrome. Second, when a patient simultaneously presents with rhabdomyolysis, acute kidney injury, and distributive shock with generalized edema, crush syndrome complicated by capillary leak syndrome should be strongly suspected rather than simple hypovolemic or cardiogenic shock. A negative central venous pressure is an important objective finding supporting this diagnosis. Third, the treatment strategy requires a fundamental shift: avoid large volume crystalloid resuscitation, prioritize vasopressors to maintain perfusion pressure, and start continuous renal replacement therapy early rather than waiting for traditional dialysis indications. Even when a negative central venous pressure suggests severe hypovolemia, large volume fluid resuscitation should not be performed. Fourth, hypostatic pneumonia must be managed aggressively as an important component of the systemic inflammatory response, with early antibiotic use and effective airway management being crucial. Fifth, multidisciplinary collaboration is key to improving outcomes, as no single speciality can manage such a complex clinical situation alone. Sixth, skin findings such as tension bullae must be managed appropriately as part of the systemic inflammation, with the focus on preventing infection and avoiding unnecessary trauma. Seventh, even after an extremely critical illness and severe neurological deficits such as complete hemiplegia, good functional recovery is possible with timely, appropriate treatment and subsequent rehabilitation. Standardized post-discharge rehabilitation is essential.

This study has several limitations. This is a single case, so the treatment strategy was individualized. Although the finding of a negative central venous pressure had important clinical significance, its rarity prevents systematic quantitative analysis. The lack of more advanced hemodynamic monitoring such as invasive arterial blood pressure waveform analysis or cardiac output monitoring limited our ability to fully assess the hemodynamic status. Because the patient was sedated and comatose, some neurological examinations and pain related assessments could not be completed, and detailed evaluation of lower limb sensation and motor function was limited. Follow up after discharge was conducted only by telephone, lacking in-person neurological assessment and follow up imaging. Future larger case series are needed to validate the treatment strategies summarized in this case.

## Conclusion

For patients suspected of having an acute stroke and found lying on the ground, clinicians must look beyond the brain. This case reveals a unique high-risk clinical scenario where prolonged immobilization after intracerebral hemorrhage can lead to crush syndrome and secondary capillary leak syndrome, forming a deadly triad. This combination necessitates a paradigm shift from neurocentric care to systemic resuscitation. Our experience demonstrates that even with severe initial deficits, favorable functional outcomes are achievable through timely multidisciplinary intervention.

## Data Availability

The original contributions presented in the study are included in the article/supplementary material, further inquiries can be directed to the corresponding author.
